# Functional links between thermoregulation and sleep in children with neurodevelopmental and chronic health conditions

**DOI:** 10.3389/fpsyt.2022.866951

**Published:** 2022-11-14

**Authors:** Susan M. McCabe, Chris R. Abbiss, Jean-Pierre Libert, Véronique Bach

**Affiliations:** ^1^School of Medical and Health Sciences, Edith Cowan University, Joondalup, WA, Australia; ^2^PeriTox UMR_I 01, University of Picardie Jules Verne, Amiens, France

**Keywords:** thermoregulation, sleep, vigilance, children, disability, chronic health conditions

## Abstract

The bi-directional relationship between sleep and wake is recognized as important for all children. It is particularly consequential for children who have neurodevelopmental disorders (NDDs) or health conditions which challenge their sleep and biological rhythms, and their ability to maintain rhythms of participation in everyday activities. There are many studies which report the diverse reasons for disruption to sleep in these populations. Predominantly, there is focus on respiratory, pharmaceutical, and behavioral approaches to management. There is, however, little exploration and explanation of the important effects of body thermoregulation on children’s sleep-wake patterns, and associated behaviors. Circadian patterns of sleep-wake are dependent on patterns of body temperature change, large enough to induce sleep preparedness but remaining within a range to avoid sleep disturbances when active thermoregulatory responses against heat or cold are elicited (to maintain thermoneutrality). Additionally, the subjective notion of thermal comfort (which coincides with the objective concept of thermoneutrality) is of interest as part of general comfort and associated behavioral responses for sleep onset and maintenance. Children’s thermoregulation and thermal comfort are affected by diverse biological functions, as well as their participation in everyday activities, within their everyday environments. Hence, the aforementioned populations are additionally vulnerable to disruption of their thermoregulatory system and their capacity for balance of sleep and wakefulness. The purpose of this paper is to present hitherto overlooked information, for consideration by researchers and clinicians toward determining assessment and intervention approaches to support children’s thermoregulation functions and promote their subjective thermal comfort, for improved regulation of their sleep and wake functions.

## Introduction

Sleep and thermoregulation are critical biological functions. Through dynamic physiological mechanisms, the functions of both are affected by behavioral, social, and environmental factors, including circadian rhythms of meals, exercise, bathing, indoor and outdoor activity, and associated exposure to ambient temperatures and light (1). They are tightly related, and have direct impacts on each other (2–4). The association between these functions and the impact of biological, behavioral, social and environmental factors is well understood in the general adult population, allowing for strategies to alter thermoregulation and improve sleep onset, maintenance and quality, and to optimize the patterns of sleep, wakefulness and daytime performance (5–8). However, thermoregulation and sleep is less well understood in children, especially those with neurodevelopmental disorders (NDDs) or chronic health conditions (CHCs).

All children need the appropriate quality and duration of sleep, for optimal physical and mental health (9, 10), learning and behavior (11–13), and meaningful participation in their daily lives (14). Sleep is especially important for children with NDDs and CHCs who, along with their caregivers, are vulnerable to additional challenges to their health, participation and wellbeing (15, 16). Indeed, sleep has valuable therapeutic potential for these children (17, 18). Unfortunately, it is common for these children to have difficulties with sleep, and with their patterns of sleep and wake (19–22). Furthermore, they are more susceptible than their peers to thermoregulation difficulties. Thermoregulatory dysfunction is discussed by Svedberg et al. (23) who found a higher incidence of cold extremities in children with severe neurological impairment, compared to their peers, with skin temperatures in the feet significantly lower in the non-ambulant children than those who walked. More specifically, difficulties with thermoregulation during sleep are a concern for these children (24, 25). Indeed, a recent study of 33 children with cerebral palsy (CP), found that 37.5% of children had sleep hyperhydrosis, compared to 4.2% of the control group of typically developing children (26). Similarly, a retrospective study of sleep concerns in 154 children with cerebral palsy, aged 1–18, found that approximately 33% reported temperature and perspiration as major concerns affecting their sleep (27). This was the case across all age groups (aged 1–6, 7–12, 13–18 years). Despite these known difficulties, and the extensive knowledge on the interaction between sleep and thermoregulation, there is no published evidence to guide research and practice for management of sleep and wake in children who have NDDs and CHCs and thermoregulation difficulties.

The dynamic and multi-directional relationships between the biological, behavioral, social, and environmental factors that affect sleep and thermoregulation can be understood when viewed within the framework of the International Classification for Functioning, Disability and Health for Children and Youth [ICF-CY; (28)]. This well-established framework represents the dynamic interaction of biopsychosocial components (body functions and structures, activity and participation, environments and other contextual factors) which influence the determinants of health and wellbeing and the long-term consequences of living with a CHCs or disability. Most importantly, it guides the focus of research and clinical practice toward promoting children’s participation (their active engagement in the important and meaningful aspects of their lives) by illustrating the variability of functioning within everyday settings and noting effects of environments and personal factors such as gender, age, ethnicity, social and educational background, behavior patterns, and life events (29).

This paper is aimed at bringing attention to clinicians and researchers who work to support sleep of children with NDDs and CHCs, of the functional relationships between sleep and thermoregulation, and to postulate pathways to optimize this important aspect of participation and quality of life in these children.

## Thermoregulation

Children and adults are homeotherms. Through dynamic physiological and behavioral thermoregulation functions they typically maintain a constant resting core body temperature between 36.5^°^C and 37.5^°^C despite changes in the surrounding environment. Thermoregulation is a critical biological function, for maintenance of vital physiological conditions for cell function, systems function and life itself (4). When conditions or environments challenge the thermoneutral state, biological, and behavioral responses are elicited, with effects on other homeostatic systems (hormonal, digestive, cardio-vascular, respiratory) and alterations to behavioral states (sleep, appetite, psychological stress, vigilance, performance) and wellbeing (30).

The body system for thermoregulation is described by two compartments: a core (including organs such as the lungs, heart, abdominal organs and brain) and a peripheral “shell,” corresponding to skin layers and associated musculo-skeletal, nervous and circulatory systems. The core system has a relatively stable temperature, and is regulated and maintained by a combination of feedforward and feedback mechanisms (4). Feedback responses occur in response to changes in internal temperatures which are detected by thermoreceptors in the core organs, and are triggered when the core temperature deviates from the homeostatic range. The peripheral “shell” is responsible for feedforward mechanisms—pre-emptive responses to anticipated thermal challenges, which are triggered prior to change in core temperatures, primarily through functions of cold and warm thermoreceptors in the skin. These dynamic interactions are heavily controlled by the autonomic nervous system (ANS), with integration mainly at the suprachiasmatic nuclei (SCN) in the pre-optic area of the anterior hypothalamus [see (31) for analysis of skin temperatures as feedforward or feedback systems].

**FIGURE 1:**
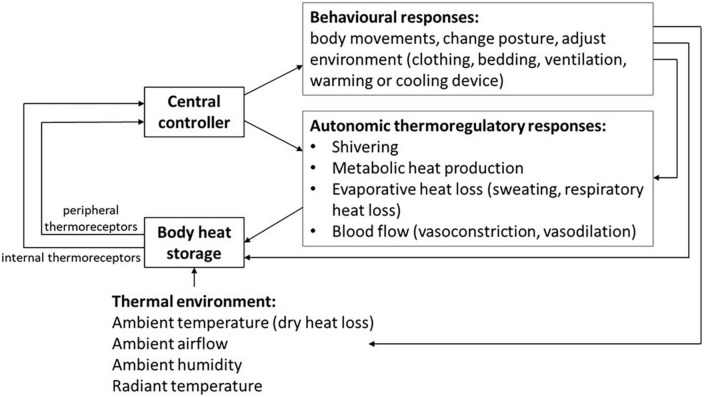
Autonomic and behavioral thermoregulatory response systems for homeostasis in varied thermal environments.

Further to this, thermoregulatory responses can be described through a model which includes a passive system (represented by heat exchanges between the body and the environment) and an active controlled system of thermosensors, central controller and effector mechanisms for thermogenesis (heat production), sudomotion (activity of sweat glands), behavior (changes in posture and movement, and adjusting the environment, clothing or bedding), and vasomotion (vasodilation and vasoconstriction) (Figure 1). In environments with ambient temperature ranges that are “thermoneutral” an almost constant and normal core body temperature is maintained, through autonomic changes in the peripheral skin blood flow (influencing the thickness of the shell compartment), with minimal metabolic heat production. Both core and skin temperatures are controlled *via* these homeostatic responses, with skin temperature involved in the regulating of core temperature (4). Outside the boundaries of the thermoneutral zone, active thermoregulatory responses are required to maintain homeothermia. Thermoregulatory responses to cold environments involve vasoconstriction, shivering and increased body activity, whereas in warm environments vasodilation and sweating occur. When these responses are not sufficient to compensate for the external thermal challenge, core body temperature decreases or increases and homeothermia is lost. Thus, constant core body temperature is a process of homeostasis which results from the body’s balance between heat production and heat losses in association with behavioral and environmental factors.

Such is the complexity of thermoregulation, and the dynamic interaction between the body and environments, it can be viewed through specific models of function of the nervous system (4, 32), or through multi-element models as reviewed by Katic et al. (33), who report 22 different thermophysiological models through the years 1970–2016. Additional complexity emerges when we consider the notion of thermal comfort, and the influence of age, and sex.

Thermal comfort is described as the result of combination and adaptation of factors of the body in context with the environment (34) and can be measured through parameters of subjective comfort sensations, as well as measures of skin temperatures and sweat rate as functions of metabolic rate. It can be used to determine optimal (or otherwise) factors for function in occupational and domestic settings, and accounts for effects of ambient temperatures and airflow, and effects of various types of materials used in buildings, bedding and clothing (35). Additional factors are important to thermal comfort, including acclimatization to natural environments, habituation to different climate zones (for example time spent indoors in air-conditioning vs. the dynamic conditions of naturally ventilated environments), seasonal adaptations, diurnal rhythms and, interestingly, the effects of perceived personal control, light intensity, and personality traits (36).

Thermoregulation and thermal comfort vary according to age and gender. The control of environmental temperature is particularly important in children, since their body surface relative to body mass is typically greater than adults. Thin body segments (arms and legs), and lean body shape are subjected to larger and more rapid heat exchanges with the environment than thicker body segments or larger body shape (37). Interestingly, Inoue et al. (38) found that when the air temperature is lower than skin temperature, prepubertal children can thermoregulate as efficiently as young adults, due to their greater surface area-to-mass ratio and relatively greater heat loss from cutaneous vasodilation on the head and trunk. In contrast, in their study comparing thermoregulatory responses of pre-pubertal boys to young men to exposure to linear increases in air temperature, Inoue et al. (39) found that when heat stress was increased the mean body temperature at the onset of sweating was significantly greater in the boys than in the men. They concluded that compared with young men, prepubertal boys manifest greater physiological and perceptual strain under heat stress when air temperature exceeded skin temperature. Reflecting this, in their review of the literature on human thermal comfort in the built environment, Rupp et al. (40) report that young children are shown to have preference for lower temperatures than adults, with apparently greater sensitivity to changes in their metabolism. In the same review, the authors also reported on the gender differences in thermal sensation between school aged girls (more sensitive to low temperatures) and boys (more sensitive to high temperatures).

## Thermoregulation and sleep

### Patterns of body temperatures and sleep

The fundamental relationship between the circadian patterns of body temperature change and sleep-wake is well known, with clear patterns of change in the sensitivity and functions of the thermoregulatory system across the sleep-wake cycle and across stages of sleep. Sleep propensity is associated with an observed drop in core body temperature (T_core)_. Correspondingly, readiness for wake is accompanied by a rise in T_core_. Interestingly, this pattern of rise and fall of core body temperature across the 24 h sleep-wake period is the inverse of patterns of change in melatonin (41). The drop in T_core_ that triggers sleep onset is precipitated by rise in distal skin temperatures (T_distal_), namely at the extremities (hands and feet). This commences approximately 100 min before sleep onset and is known as “vegetative sleep preparedness,” first described by Magnussen in 1938 [cited in van den Heuvel et al. (42)] The rise in T_distal_ is caused by peripheral vasodilation, allowing inflow of heated blood from the core to the shell, and facilitating heat loss to the environment through the small peripheral blood vessels, the arterio-venous anastomoses (AVAs) which are particularly abundant in the glabrous (non-hairy) palms of the hands, soles of the feet, surface of the ears, and certain facial sites (31, 43). The degree of peripheral vasodilation can be estimated by the difference between T_distal_ and proximal skin temperatures (T_proximal_, variously measured at thighs, abdomen, shoulders and back). Importantly, this difference, T_distal_ minus T_proximal_, the distal-proximal gradient (DPG), is regarded as the most significant thermal marker and mechanism of sleep readiness. Indeed, Kräuchi et al. [(44), p.36] wrote that “the degree of dilation of blood vessels in the skin of the hands and feet, which increases heat loss at these extremities, is the best physiological predictor for the rapid onset of sleep.”

The relationship between distal skin vasodilation (with rapid rise in T_distal_ and DPG) and sleep onset is understood to be causal, with several studies performed in adults showing that experimentally induced distal vasodilation by subtle skin warming promotes sleep. Accordingly, skin warming for passive body heating (e.g., shower or bath before bedtime) or non-thermal manipulations capable of promoting distal vasodilation (e.g., lights off, lying down, a spicy meal, physical exercise) may increase sleepiness, accelerate the process of falling asleep and improve sleep maintenance in adults (45–48). Interestingly, a recent study of the effects of manipulation of periocular skin temperatures of 19 healthy males showed that a warming eye mask, used prior to sleep, significantly increased the temperatures of hands, feet and the rise of DPG, with significant increase in self-reported sleepiness (49). Most recently, in their randomized controlled study of 11 young healthy males, Haghayegh et al. (50) found that selective thermal stimulation, consisting of a heated pillow that provided mild heating to the cervical spinal skin, in combination with a cool central and warm peripheral temperature-controlled mattress, induced significantly greater distal vasodilation, increased rate of change of DPG, shorter sleep onset latency and significantly better subjective sleep quality in the treatment than the control nights.

Behaviors that lead to pre-sleep relaxation and reduced anxiety when retiring for bed also promote skin vasodilation prior to sleep onset by decreasing sympathetic nervous system activity (51). Additionally, the hypnotic effects of medications such as benzodiazepine and temazepam, and the sleep readiness effect of melatonin is associated with their effects on distal skin vasodilation (52, 53). In contrast, adults with conditions or behaviors which cause attenuated rise in T_distal_ take longer to fall asleep (8, 48). This important relationship between rapid rise in T_distal_ and DPG and sleep onset has also been shown for preterm neonates, infants, and school-aged children (54–56). Logically, the propensity for morning wake correlates with core temperature rise in the morning, as a result of distal skin vasoconstriction which is evident in lowering distal skin temperatures and DPG. Consistent with this, manipulations that induce distal vasoconstriction promote awakening and vigilance in adults (57). Similar studies have not been reported for children.

Consideration of T_proximal_ is also important to understanding sleep, especially in relation to sleep quality and maintenance. This may be especially important for young children. In their study of sleep and skin temperatures of pre-school children and their mothers, Okamoto-Mizuno et al. (58) found that children’s proximal temperatures increased more than distal temperatures, and that heat dissipation in this group was dependent more on increase in T_proximal_ than T_distal_. They noted cardiovascular differences in this younger group, and surmised that T_proximal_ is nearer to the body core and possibly of more benefit for heat loss and decreased cardiovascular strain than the more distal sites. Interestingly, this group of young children were noted to predominantly sleep without bed coverings, and to move about the bed surface during sleep, enabling a greater dry-heat exchange during sleep and indicating a greater dependence on behavioral thermoregulation than for adults. While many studies report T_abdomen_ as a measure of T_proximal_, we have recently demonstrated that T_back_ was a good indicator of T_proximal_ during sleep of school aged children (59). This corresponds with studies of adults, which report that conductive heat loss through the proximal back is critical for slow wave sleep and subjective sleep quality (60, 61). Similarly, Lan et al. (62) found that local cooling of T_back_ was most effective in alleviating thermal stress in a warm environment. Less frequently reported than T_distal_ and T_proximal_, forehead skin temperature (T_forehead_) is also considered to be an important region of thermoregulation (63, 64). It is reported to be affected by the temperature of the underlying brain, and to have a high rate of heat transfer with ambient temperature due to venous as well as arterial systems, making it a unique site of T_skin_ measurement (31). In studies of adults and elderly, T_forehead_ has been shown to be particularly affected by seasonal ambient temperatures (65), and may be an important factor in sleep maintenance within this context.

While there is a wealth of research examining the association between body temperature and sleep in adults, it is not clear if such findings can be directly translatable to children, thus warranting further research in this population.

### Thermal comfort and sleep

The notion of thermal comfort is fundamental to understanding the interactions between thermoregulation and sleep. Warm or cool challenges (beyond thermoneutral) before and during sleep affect the timing of sleep onset, the duration of sleep stages and the overall efficiency of sleep (66, 67). They also affect subjective perceptions of “thermal comfort” and elicit associated behavioral thermoregulation strategies such as adjusting bedding and changing body position, affecting sleep depth and propensity to wakefulness (8, 48, 68, 69). While autonomic responses to hot or cold stimuli are reduced during REM compared with NREM sleep, REM sleep is more vulnerable to thermal discomfort than the other sleep stages (70).

Thermal comfort during sleep is underpinned by physiological and behavioral responses to environmental factors: seasonal, household and bedroom temperature and humidity, and, more specifically, the “microclimate” of the bed which is influenced by interactions of bedding and clothing. These factors are affected by building design and the use of heaters, air-conditioners, fans and ventilation (70). In their review of the environmental parameters for optimal sleep, Caddick et al. (71) recommended ambient bedroom temperatures between 17^°^C and 28^°^C, depending on effects of bedding and with relative humidity between 40 and 60%. The effects of environmental temperatures have been found to vary across sleep periods. Whilst humid heat is reported to particularly affect slow wave sleep in the earlier phase of sleep period, cold exposure is found to impact on quality of sleep in later segments of sleep (69). Furthermore, in their review study, Lan et al. (62) reported that a cooler sleep environment at the beginning of the sleep period caused delayed sleep onset, while in a warm setting, local cooling to neck and back improved thermal comfort and sleep efficiency during the sleep period. Associated with this, the microclimate of the bed is particularly important for thermoregulation and sleep, for sleep onset and protection of sleep stage structure (70–72). In their review of thermal environment and sleep quality, Lan et al. (70) found that an in-bed microclimate of around 30^°^C was most consistently associated with thermoneutrality for the sleeping human body, with relatively small variation across seasons and change in ambient temperatures. Various studies in adults have shown that the microclimate can be determined and indeed manipulated by types of mattresses (60, 61, 73–75), bed sheets (76), personal heating or cooling devices such as electric blankets or airflow devices (77–80) and clothing worn during sleep (46, 81). While environment conditions, bedding and the microclimate clearly influence body temperature and perspiration, there is limited research examining the thermal comfort of children within their normal sleeping environments.

## Thermoregulation and sleep for children with neurodevelopmental and chronic health conditions

**FIGURE 2:**
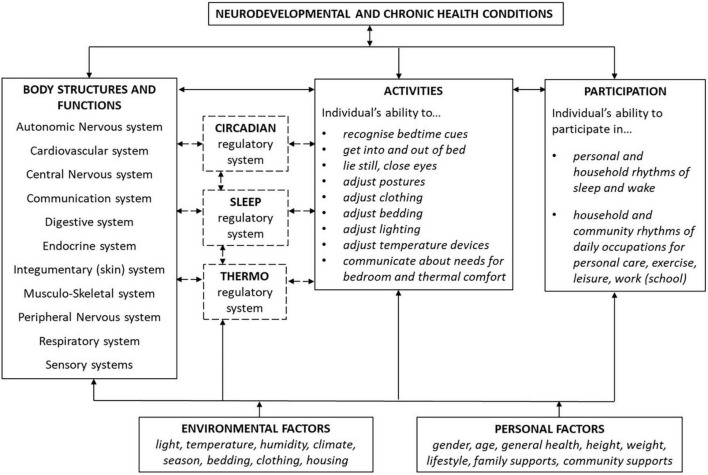
Thermoregulation and sleep in context of the ICF.

The complex and interactive functions and effects of thermoregulation and sleep are especially important when considered in the contexts of everyday living. This is relevant for people of all ages, in all environments, with varied daily occupations, and with various health and medical conditions. For children with NDDs and CHCs, the dynamic functional relationships between these factors can be best understood when viewed in context of the framework of the ICF-CY. The following sections will discuss the interactions between thermoregulation and sleep in relation to body structures and functions, activity and participation, environments and personal factors, as illustrated within the adapted model of the framework of the ICF-CY (Figure 2).

### Children’s body structures and functions affect their thermoregulation and sleep

With the understanding that central and peripheral interactions of the ANS involve all body structures and functions, and knowledge of the potent interaction of behavioral and autonomic thermoregulatory responses for thermal homeostasis and for sleep, it is clear that disturbance to structures (anatomy) or the functions (physiology) of body systems can have important implications for thermoregulation and sleep in children with NDDs and CHCs.

For children with NDDs, neurological impairment may occur at cortical and subcortical levels, with direct impact on thermoregulatory and sleep system functions. Children with neuro-motor conditions such as cerebral palsy commonly experience pain, circadian dysregulation and hyper-arousal in association with co-morbid epilepsy, and impairment of their respiratory, cardio-vascular, endocrine, gastro-intestinal, integumentary, musculo-skeletal and sensory functions, with impact on their ANS and associated thermoregulatory and sleep system functions (82, 83). Additionally, vision impairment is also common in this group (84, 85), impacting the SCN pathway of effects of light and dark on circadian functions of thermoregulation, and sleep and arousal. The functional relationships between hormonal, sympathetic and parasympathetic pathways mean that children with developmental conditions such as autism spectrum disorder (ASD), attention-deficit/hyperactivity disorder (ADHD) and fetal alcohol spectrum disorders (FASD) also have ANS dysfunction (86–88). Furthermore, pain and discomfort are common for these children, related to co-morbid anxiety and gastro-intestinal, musculo-skeletal or sensory regulation impairment, compounding disruption to the functional relationship between their thermoregulation and sleep (89).

Sleep disruption is also widely reported in children with CHCs, due to impairment of their body structures and functions, and, associated pain and discomfort, with effects on ANS function and rhythms of thermoregulation, thermal comfort and sleep. Given the importance of skin in thermoregulation, conditions which involve impairment of skin function are of particular interest. Sleep disruptions in children with eczema are commonly reported (90, 91), and the association between skin conditions, thermoregulation and sleep is well described by Gupta and Gupta (92). Sleep disturbance is also commonly reported in people with other conditions which affect skin and peripheral vascular functions, such as burns injury (93, 94), diabetes (95), obesity (96) and connective tissue disorders such as Ehlers Danlos syndrome (97). Individuals with childhood cancer are also reported to experience sleep difficulties (98, 99), with hyperarousal due to pain or anxiety. There is a logical relationship between the effects of these conditions on ANS, thermoregulatory functions and sleep.

There is potential for remediation of sleep and thermoregulation difficulties through intervention at the level of body structures and functions. Children who have NDDs and CHCs and need medications, procedures and hospitalization are likely to experience various levels of pain and anxiety, with effects on ANS and vasomotor functions, and subsequent impact on the patterns of core and peripheral temperature changes that are essential to circadian patterns of sleep and wake. With consideration of the individual responses to interventions, these effects could be mitigated by consideration of the timing and dosage of medications (effects on pain and anxiety; effects on core and proximal temperatures; effects on distal vasodilation) and consideration of timing of medical appointments and procedures. Furthermore, changes in environmental conditions, and types and timing of activities may moderate the physiological functions and responses at the level of the ANS, with impact on thermoregulatory effects on sleep, as discussed below.

### Children’s activity and participation affect thermoregulation and sleep

The timing and rhythms of activities and behaviors play an important part in circadian biological function, with interactions that affect and are affected by thermoregulation and sleep. van den Heuvel et al. (42) confirmed the importance of regularity in bedtime time and demonstrated that physiological sleep preparedness (i.e., distal vasodilation, with rise in T_distal_, rise in DPG, drop in T_core_) was associated with habitual activities for sleep onset. In contrast, when activities associated with sleep onset varied, distal vasodilation was attenuated. Supporting this, Martinez-Nicolas et al. (100) showed that people with the highest contrast between their day and night factors of activity, body position and light exposure had more marked circadian rhythms of T_distal_ and sleep. The authors postulated that intensifying the contrast between day and night lifestyle factors may enhance the rhythm of the circadian system. It is notable that pre-sleep activities (anticipating sleep, assuming supine position, cognitive and physical relaxation, switching off the light) can enhance distal vasodilation and promote sleep onset. It is common for children with NDDs and CHCs to experience disruptions to pre-sleep activities, due to the need for complex personal and medical care prior to sleep, as well as communication, cognitive, sensory or behavioral difficulties which impact their ability to anticipate sleep and readily engage in suitable pre-sleep activities (101). There is a likely compounding effect to this, with a bidirectional relationship between parenting stress and inconsistent bedtime routines (102).

More specifically, circadian changes in motor activity promote temperature changes that are associated with patterns of sleep and vigilance (103). This is important when considering the effects of movement impairment on children with neuro-motor conditions, who may have consistently limited or disrupted activity level across any 24-h period (104–106), as well as those with developmental conditions and associated dysregulation of their levels of activity and arousal behaviors (107, 108). For sleep onset, there is an additional important effect of postural changes on ANS and thermoregulatory responses when lying down, unloading the cardiovascular system and reducing sympathetic tone (8). Children with neuro-motor conditions may need to be supported in an upright position during and following their feeding regime, or to assist with breathing throughout the sleep period (109, 110) creating an additional impediment to the vasomotor process that is essential to sleep onset and maintenance.

The timing and temperature of bathing or showering have been shown to influence the rhythms of thermoregulation and sleep. In particular, studies have shown that warm bath, shower or foot bath in the hour before sleep enhance distal vasodilation, with increased rate of change in DPG and reduced sleep onset latency (45). This is significant for families of children with NDDs and CHCs. Some may need to provide bathing in the mornings, for necessary morning hygiene, or need for a simpler evening routine or because of behavioral or sensory responses which cause them to be increasingly agitated or excited during bath or shower, and thus less able to calm and settled for sleep. Some may simply be unaware of the potentially powerful physiological effects of warm bath or shower before sleep and thus miss the benefits of this routine practice.

The type timing, volume, and stimulant consumption and micronutrient intake food and drink intake is known to directly influence sleep (111). Additionally, diet may also influence sleep though effects on body temperature regulation. Indeed, it is known that various foods have vasomotion effects, such as spicy foods, and those high in nitrates or caffeine (112, 113). Additionally, Fronczek et al. (6) showed that the temperature of food or drink may directly affect thermoregulation and vigilance. Children with NDDs and CHCs may have significant disruption to their intake of food and drink. Those with neuro-motor conditions such as cerebral palsy may have disruption to their oral-motor and gastro-intestinal functions, needing modified diets, or overnight tube feeds (oro-gastric, naso-gastric or percutaneous endoscopic gastrostomy), with disturbance to the usual rhythms and timing of oral intake (114). Those with neurodevelopmental conditions such as autism, ADHD or intellectual disabilities may have sensitivities or aversion to specific food types, being selective about or averse to tastes, textures and temperatures of food and drink (115). The type, amount, temperature and timing of food and drink intake may also be compromised for children with CHCs such as diabetes, and intolerances or allergies (116–118).

### Children’s environments affect thermoregulation and sleep

The seasonal effects of temperature, humidity and light on sleep onset, maintenance and architecture are widely reported, with variations according to geographical locations, age, occupations, gender, and health conditions. These factors interact with building design and the use of heaters, air-conditioners, fans and ventilation, with varied impact on temperature and humidity of the ambient sleep setting (70). More specifically, the creation of an approximately thermoneutral microclimate of the bed is increasingly recognized as particularly important for thermoregulation and sleep, for sleep onset and protection of sleep stage structure, as described above. With the interactive effect of the ambient room environment, the microclimate of the bed is affected by materials used in the mattress, pillows, bed linen and clothing, and by presence or absence of other bodies (people or pets) in the bed (54, 76, 119, 120). The increasingly diverse and sophisticated range of types and materials used for bedding and clothing, such as blankets or throws with timers and varied heating zones, and the development of “smart” materials such as phase change materials, reflects the prevailing knowledge about the importance of thermal comfort for sleep (121). Clothing insulation is a relevant component of behavioral thermoregulation for management of the sleep microclimate (120, 122). Depending on the season, cultural preferences and age, children may or may not wear clothing (pajamas) in bed. Clothing may be especially effective in reducing heat loss, but when used with bedcovers and in warm settings, can increase the risk of body overheating. The ability to choose and adjust bedding and clothing is an important, dynamic behavioral response toward thermoneutrality and thermal comfort, however, children with impairment of their movement, sensory or cognitive functions may have reduced or no capacity for this.

In addition to the above factors, it is important to consider the impact of timing, type and intensity of ambient light on the rhythms of sleep and thermoregulation. There is synchrony between the strength of circadian rhythmicity and the timing of evening dim light and morning bright light. Evening exposure to bright light will reduce evening sleep propensity, while morning exposure to bright light will increase evening sleep propensity (123), and fragmentation in the rhythms of light and dark exposure is associated with more fragmented sleep (124). Even low light levels during sleep, with eyes closed, can disrupt circadian responses (125). Through interactions at the SCN, light in the evening can reduce melatonin secretion, with associated slowing of rise in T_distal_ and delay in decline in T_core_ (126). In children, the deleterious effects on sleep of portable electronic devices in the bedroom setting are particularly notable (127). The disruptive effects of light on circadian functions of sleep and thermoregulation varies with children’s age, as described in the review by Logan and McClung (128), of circadian changes across the lifespan.

The impacts of environmental factors on sleep and thermoregulation in children with NDDs and CHCs is important for many reasons. Children with mobility impairment may be unable to manage their environment (e.g., open or close windows, or adjust their bedding or clothing) as needed. Those with sensory and cognitive impairment may not register the sensation of thermal discomfort, and be unable to make the necessary behavioral adjustments to promote their thermal comfort. Moreover, those with conditions such as autism or ADHD may have sensory preferences which cause them to actively eschew the use of footwear before bedtime, with resulting cold feet and attenuation of the distal vasodilation which is essential to sleep onset. Those with communication impairment may be unable to communicate their comfort needs to their care providers so that the environment is adjusted to best suit them. Additionally, those with communication impairment may rely on electronic devices and use of screens for communication function at bedtime and possibly during occasions of night waking. Furthermore, the child’s condition may require instruments or actions which compromise the optimal thermal environment. For some, it is necessary to maintain bedroom lighting, for safe provision of nighttime care and use of technology to support sleep (129). Children with movement or postural impairments require positioning equipment such as padded brackets or customized cushions to support their body shape (130), with the encompassing effect of foam and padding materials likely to cause a warmer microclimate in the bed. Those with incontinence may need moisture proof bedding for hygiene purposes (27), compromising attempts to use thermobalancing bedding and clothing materials for optimal management of the bed microclimate.

### Children’s personal factors affect thermoregulation and sleep

There are numerous reports of the effects of personal factors on children’s sleep. Variously, these factors can be understood to also impact on thermoregulation, although the connection is rarely made clear. In a recent study of the impact of gender differences on the sleep of adolescents (131), it was shown that sleep quality and daytime dysfunction was significantly worse in girls than in boys. For girls, reduced sleep duration was particularly associated with consumption of hot drinks before bedtime while for boys the key factor was time spent on technology. Body mass index (BMI) is also an important factor, with U-shaped relationships reported between BMI *z*-scores and poor sleep quality. These factors are found to be related to family and household function, with household “chaos” reported to be associated with less physical activity, less sleep and more screen time in households that have elevated stimulation, lack of structure and reduced predictability in activities and routines (132–134).

Children with NDDs and CHCs, and their families, are additionally vulnerable to the effects of personal factors, with impact on related factors which affect their sleep and thermoregulation. A particular concern is the effect of caring for a child with high support needs on caregiver health and wellbeing. Poor child sleep has a strong association with poor parental sleep, with associated impairment in physical and mental health, including an increased risk of cardiovascular and metabolic disease and a weakened immune system (135, 136).

## Discussion

The important interaction between sleep and thermoregulation is well known, as is the fact that children with NDDs and CHCs are more vulnerable than their peers to sleep disturbance and the associated deleterious effects. There is a dearth of studies reporting on thermoregulation and sleep in these groups. It is likely that this is because the questions have not been asked and studies not been done, rather than through absence of an important relationship. Despite the prevailing knowledge about the importance of distal temperatures in relation to sleep onset and maintenance, to our knowledge, there are no standardized or validated pediatric sleep questionnaires which ask about children’s temperatures before and during the sleep period. In their recent overview of 70 pediatric sleep tools, Sen and Spruyt (137), describe an extensive range of conditions which are considered, including sleep disordered breathing, morning symptoms, nighttime awakenings, insomnia, excessive daytime sleepiness, daytime behavior, sleep habits and irregular/delayed sleep phase sleep routine, bedtime anxiety, morning tiredness, night arousals, sleep disordered breathing, and restlessness before and during sleep. They note that there is an emerging need for further research into children’s sleep, with tools which assess sleep ecology, routines and hygiene, regularity, and treatment. Concerning such research, it is notable that the Children’s Sleep Habits Questionnaire [CSHQ; (138)], one of the most widely used questionnaires to assess sleep problems in children, has one question which could pertain to body temperature regulation (“awakens screaming, sweating”), as part of the subscale of parasomnias. It does not include questions about children’s observed patterns of body temperature or thermal comfort before and during the sleep period. Similarly, another widely used pediatric sleep questionnaire, the Sleep Disorders Scale for Children [SDSC; (139, 140)] includes 2 questions, specifically about perspiration before sleep and during sleep, to give a score relating to functions of sweating (reported as the domain SHY, sleep hyperhydrosis), with no option to provide information about patterns of skin temperatures or thermal comfort before or during sleep. Further to this, we note, with interest, one recent study (examining sleep hygiene factors in young children with and without ASD) which includes thermal comfort variables. For this study, Richdale and Schreck (141) developed a questionnaire which included a section on Thermal Sleep Environment description, with 16 items which asked about the child’s typical bedding, sleep wear and sleep environment. Parents were asked about the use of extra stimuli to keep warm (e.g., hot water bottle, hat, socks), and if the child was too cold or hot at night in relation to warm-hot/cool-cold weather or summer/winter.

Given the clear relationship between sleep and thermoregulation, the vulnerability of children with NDDs and CHCs to difficulties with sleep and thermoregulation, and the known interplay between domains of body structure and function, activities and environments on sleep and thermoregulation, it is clear that there is broad scope for further relevant research, to guide possibly valuable clinical applications (Table 1).

**TABLE 1:** Case descriptions of children with neuromotor, developmental and chronic health conditions, illustrating the interactions of body structures and function for thermoregulation and sleep, with opportunities for therapeutic modification to activities and environments.

Child 1		
***6 yo Sam has cerebral palsy***, *with reflux, epilepsy and pain (related to muscle, joint and gastro-intestinal functions). He has severe movement impairment and cannot adjust his position, don/doff clothing or bedding or control his bedroom environment. He has a plastic mattress protector for incontinence. He has cushions in his bed, to support his body in a safe and comfortable position. He is restless and distressed before sleep. He cries out several times each night. His parents report that he is always hot and flushed, even on cold nights, with perspiration on his back and head. They are unsure how to help Sam be comfortable for sleep. They are very tired, and worried about his wellbeing.*		

Body functions affected	Activities affected	Opportunities for remediation of T^o^ factors through activities and environments

ANS, CVS, CNS, DS, M-SS, PNS, RS, SS, VS	Get in/out of bed adjust postures adjust clothing adjust bedding adjust lighting adjust T^o^ devices	Activities – warm or tepid shower or bath before bedtime Environments – socks or slippers before bedtime – high heat-capacity mattress or overlay – vapor permeable mattress protector – clothing and bedding made of thermoregulation material – temperature/airflow devices with timer settings – temperature/airflow devices with voice activation

**Child 2**		

***13 yo Hannah has autism***, *with gastro-intestinal pain, and anxiety. She has difficulty recognizing body cues of hot and cold, and has an aversion to many textures. She cannot tolerate socks or slippers. She has irregular bedtimes. Once in bed she can take 2–3 h to get to sleep. During this time, she becomes restless and agitated. She uses her iPad for calming/distracting activities. Because of delayed sleep onset she sleeps until late morning and becomes agitated when prompted to get out of bed. She frequently misses school.*		

Body functions affected	Activities affected	Opportunities for remediation of T^o^ factors through activities and environments

ANS, CVS, DS, ES, SS, VS	Recognize bedtime cues be calm before bedtime wear socks or slippers lie still, close eyes	Activities – warm or tepid shower or bath in the hour before bedtime – alternative to screens for calming/distraction before sleep Environments – heat pack in foot area of bed at bedtime – high heat capacity mattress or overlay – clothing and bedding made of thermoregulation material – open windows or doors, or cooling fans for airflow

**Child 3**		
***8 yo Eva has eczema***. *She frequently has flare-ups, causing her skin to feel hot and itchy. She finds this especially troublesome at night, and she frequently wakes and asks for parent attention. Her parents note that even when she doesn’t wake or call out, she appears very restless during sleep. She is often distressed and moody in the mornings, and needs prompts and support to eat her breakfast and get ready in time for school. Her teachers comment that she seems tired and inattentive at school.*		

Functions affected	Activities affected	Opportunities for remediation of T^o^ factors through activities and environments

ANS, CVS, IS	be calm before bedtime lie still, close eyes	Activities – tepid shower or bath in the hour before bedtime – relaxation techniques for calming before sleep Environments – socks before bedtime – high heat capacity mattress or overlay – clothing and bedding made of thermoregulation material – open windows or doors, or cooling fans for airflow

ANS, autonomic nervous system; CNS, central nervous system; CS, communication system; CVS, cardiovascular system; DS, digestive system; ES, endocrine system; IS, integumentary system; M-SS, musculo-skeletal system; PNS, peripheral nervous system; PVS, peripheral vascular system; RS, respiratory system; SS, sensory system; VS, vision system.

## Conclusion

The purpose of this translational review is to draw attention to the functional links between sleep and thermoregulation, and to highlight the important implications for the health and wellbeing of children with neurodevelopmental and CHCs. Currently, there are missing links between the knowledge that exists regarding the importance of various aspects of sleep (onset, maintenance, architecture, rhythms, subjective quality, daytime sleepiness) for diverse pediatric populations, and the knowledge that exists regarding thermoregulation and thermal comfort in relation to these same aspects of sleep. When viewed in context of body structures and functions, activities and participation, and environment, there is seemingly boundless scope for targeted research, to promote understanding about practical, ecological assessment of thermoregulatory functions, and related interventions to support good sleep in these vulnerable populations.

## Data availability statement

The original contributions presented in this study are included in the article/supplementary material, further inquiries can be directed to the corresponding author/s.

## Author contributions

SM and VB conceptualized, drafted, contributed, modeled the manuscript, and developed the models. CA and J-PL contributed to the manuscript. All authors have reviewed and approved the final draft.
